# Association of the Apolipoprotein A5 Gene −1131T>C Polymorphism with Serum Lipids in Korean Subjects: Impact of Sasang Constitution

**DOI:** 10.1155/2012/598394

**Published:** 2011-08-16

**Authors:** Kwang Hoon Song, Sung-Gon Yu, Seongwon Cha, Jong Yeol Kim

**Affiliations:** Division of Constitutional Medicine Research, Korea Institute of Oriental Medicine, 483 Exporo, Yuseong-gu, Daejeon 305811, Republic of Korea

## Abstract

Apolipoprotein A5 (*APOA5*) was identified as a strong modulator of serum lipids. Moreover, an *APOA5* gene −1131T>C polymorphism has been associated with serum lipids, but the results are inconsistent according to ethnic and racial groups. We have genotyped and analyzed 1,619 outpatients of Korean oriental medicine hospitals who were classified into three Sasang constitution groups (SCGs), So-Yang (SY), So-Eum (SE), and Tae-Eum (TE). There were no significant difference in the distribution of the *APOA5* −1131T>C genotype among the three SCGs. Subjects with the C allele in SY and TE showed significantly lower serum high-density lipoprotein cholesterol (HDL-C) and higher triglyceride (TG) levels than noncarriers of the C allele. These results show the differences in the prevalence of decreasing serum HDL-C and elevating serum TG levels along with *APOA5* −1131T>C polymorphism according to SCG and suggest that SCG may act as a significant risk factor for hypo-HDL-C-emia and hypertriglyceridemia susceptibility.

## 1. Introduction

Cardiovascular disease (CVD) is one of the most common conventional causes of death in industrialized countries, and elevation of triglycerides (TG) levels, hypertriglyceridemia, has been shown to be related to an increased risk of CVD [1–5]. Serum TG levels vary among individuals due to both environmental and genetic factors [6, 7].

The identification of specific genetic determinants of plasma triglyceride concentrations has been attempted, and recently a novel member of the apolipoprotein family, human apolipoprotein A5 (*APOA5*), was identified by the comparative sequencing of the *APOA1/C3/A4* gene cluster region [8]. Functional studies of *ApoA5* with mice that were genetically modified to either overexpress or lack *ApoA5* provided direct evidence that ApoA5 plays an important role in plasma triglyceride metabolism. The plasma TG levels in mice overexpressing a human *APOA5* transgene were one-third those in control mice and increased fourfold in mice lacking *ApoA5*; overexpression of *APOA5* in mice using adenovirus markedly decreased (−70%) serum triglyceride levels [8, 9]. Moreover, three single-nucleotide polymorphisms, including −1131T/C, IVS3+476G>A, and 1259T/C, across the *APOA5* locus to be significantly associated with plasma TG levels were identified [8]. Specifically, an SNP −1131T>C (rs662799) in the promoter region of the *APOA5* gene has been associated with elevation of plasma TG levels in several populations of various ethnicities [8, 10–13]. In addition, several studies have demonstrated that this SNP was associated with reduced plasma high-density lipoprotein cholesterol (HDL-C) levels [13–15].

Since knowledge of genetics and molecular biology has been accumulated, interests in personalized medical care have become important in making the best therapeutic choice by facilitating predictions about drug efficacy and safety. Interestingly, Sasang constitutional medicine (SCM), a unique Korean traditional therapeutic alternative form of medicine, has already been developed with properties of personalized medicine [16]. SCM is originated from the theory that human beings are categorized into four constitutions according to the traits of an individual's temperament and body shape, and each constitution has a different drug response to certain herbal medicine and different susceptibility to pathology [16, 17]. In SCM, therefore, each constitution shares the similar aspects of bodily structure, function, and metabolism, as well as psychological and behavioral characteristics [16, 18, 19].

It has been suggested that the prevalence and relative risk of several chronic diseases were found to differ across different Sasang constitution groups (SCGs), and the same disease is often treated differently for different individuals based on the constitutionally differentiated traits in clinical practice [17]. Recently, several studies have described efforts to explain SCM from the perspective of genetics and the genome-wide scale, but much controversy still remains [20–22]. Therefore, the identification of gene variants associated with hypertriglyceridemia phenotypes according to SCG might help to individualize the prognosis, treatment, and prevention of CVD and metabolic diseases. We have recently identified that significant differences exist in serum lipid profiles among SCGs and hypothesized that some genetic factors may be responsible for this discrepancy. Therefore, the aim of the study is to evaluate the association of *APOA5* −1131T>C polymorphism with serum lipid levels in a large population-based study with Korean subjects classified by SCM.

## 2. Materials and Methods

### 2.1. Study Population

Data were retrospectively collected from 20 oriental medical hospitals in Korea between 2006 and 2009. Recruited participants were outpatients who had at least four visits. All samples and clinical information were deposited in Databank of Sasang Constitutional Medicine (DB-SCM) in the Korea Institute of Oriental Medicine. 

The SCG of an individual was determined following three procedures. Firstly, the prediagnosis of the SCG of individual was conducted by licensed medical specialist in SCM who have been in clinical practice at least 5 years. The specialist of SCM considers carefully in terms of physical body shape, appearance, temperament, and pathological symptoms of individual to prediagnose the SCG. Secondly, participant who constitutionally prediagnosed was treated with SCG-specific herbal formulae according to the individual's constitution [19, 20, 23]. We included only the patients who had been taking herbal formulae for at least 30 days and had good medication responses, showing clear improvements in the chief complaints and ordinary symptom without any adverse effects. It has been reported that the prescription which was not suitable for the SCG induced adverse reactions such as indigestion, stomachache, and evacuation troubles [24]. Finally, the SCG of individual was clinically confirmed by the specialist of SCM based on the medical chart review. 

To avoid potential confounding of the association between the *APOA5* −1131T>C polymorphism and preexisting diseases instead of SCGs, subjects with a history of CVD, hypertension, cancer, liver disease and diabetes in case report form (CVD) were excluded. Since the population of Tae-Yang is extremely low (0.03%) [25] and in present study there were only 27 participants (0.01%), we conducted analysis on three SCGs: SY, SE, and TE. Finally, a total of 1,619 patients were analyzed, including SY (*n* = 546), SE (*n* = 405), and TE (*n* = 668). 

Each subject's weight and height were measured in kilograms and in meters, respectively, and the corresponding body mass index (BMI) was calculated by dividing the weight by the square of the height. Fasting blood samples were collected via venipuncture in the morning after a fast of at least 8 hours. About 5 mL of venous blood were collected from each subject, and serum was separated for further analysis for total fasting blood glucose (FBG), total cholesterol, TG, HDL-C, and low-density lipoprotein cholesterol (LDL-C). All subjects gave written informed consent, and the ethical committee of our institution approved the study.

### 2.2. Genotyping of Single-Nucleotide Polymorphism

The genotyping of *APOA5* −1131T>C polymorphism was determined with a utilization of an unlabelled oligonucleotide probe (UOP) that spans the polymorphic nucleotide. The UOP was designed from a perfectly matched duplex with the T allele of *APOA5* −1131. Briefly, small segments of genomic sequence spanning the *APOA5* −1131T>C polymorphic site were amplified by PCR using 50 ng of genomic DNAs as a template and a set of primers (APOA5-F1  ACTCTGAGCCCCAGGAAC,  APOA5-R1  GAGTGGAGTTCAGCTTTTCC). PCR amplification was performed via a heating cycle (50°C for 2 min and 95°C for 10 min), followed by an amplifying cycle (40 cycles of 95°C for 20 seconds, 56°C for 20 seconds, and 72°C for 20 seconds) in a thermal cycler (C1000, Bio-Rad).

The PCR product was diluted with two volumes of distilled water, and the diluent was mixed with six volumes of probe solution containing 1 *μ*M of UOP, 5 *μ*M of Syto 9 (InvitrogenTM), 12.5 mM of EDTA, and 10 mM of Tris (pH 8.0) and were subsequently subjected to a thermal reaction for genotyping using a Lightcycler 2.0 instrument (Roche Diagnostics). The thermal reaction consists of a denaturation step at 95°C for 5 seconds, an annealing step at 60°C for 1 minute to allow annealing of complementary strands (UOP,  APOA5-S  AGCTTTTCCTCATGGGGCAAATCTCACTT), and a melting step with a gradual increase of temperature at a rate of 0.1°C/second until 95°C, when fluorescence emissions were read. The genotype of each PCR product was then determined from three melting patterns (major homozygote, heterozygote, and minor homozygote) based on the temperature where the corresponding UOP melts away.

### 2.3. Statistics

The quantitative variables were presented as mean ± standard deviation. The allele frequency of *APOA5* −1131T>C polymorphism was determined by gene counting. We used a Chi-square analysis to evaluate whether *APOA5* −1131T>C polymorphism was in Hardy-Weinberg equilibrium in the population. Student's *t*-test was used to analyze the difference between genders. Analysis of variance (ANOVA) was used to analyze the difference among the SCGs. Analysis of covariance (ANCOVA) is used to adjust effect of age and sex in identifying analysis for difference among the SCGs.

Multiple regression analysis was used to identify significant variables affected by genotype of *APOA5*, and effect sizes (slope) were presented as changes in minor allele carriage. Effects of sex and age are adjusted in multiple regression models. A *P*-value less than 0.05 was considered statistically significant. All statistical analyses were performed using SPSS (SPSS Inc., Chicago, IL; V14.0 for Windows).

## 3. Results

### 3.1. Clinical Characteristics of the Study Population According to *APOA5* −1131T>C Genotype

A total of 1,619 individuals, 595 males and 1024 females, were enrolled in this study. The general clinical characteristics of study subjects, including age, BMI, FBG, HDL-C, LDL-C, total cholesterol, and TG, for this study as a total and according to gender were presented in Table 1. The genotype distribution of *APOA5* −1131T>C among the 1,619 subjects analyzed was as follows: 765 subjects (269 males and 496 females) were homozygous for the T allele (TT), 712 subjects (269 males and 443 females) were heterozygous (TC), and 142 subjects (57 males and 85 females) were homozygous for the C allele (CC). The minor allele (C) frequency of *APOA5* −1131T>C was 0.305, and the genotype distributions did not deviate from the Hardy-Weinberg equilibrium.

To address the association and impact of *APOA5* −1131T>C polymorphism on the lipid profile, we evaluated the mean values of HDL-C, LDL-C, total cholesterol, and TG from each genotype and performed multiple regression analysis. The association result indicated that there was a significant association between serum HDL-C levels and the *APOA5* −1131T>C genotype (effect = −1.602 mg/dL, *P* = 3.61*E* − 04). In addition, the TC genotype group had lower serum HDL-C levels (45.76 ± 12.17 mg/dL) than the TT genotype group (46.91 ± 11.68 mg/dL), and the CC genotype group had the lowest serum HDL-C levels (43.44 ± 10.33 mg/dL). 

The association of *APOA5* −1131T>C with serum TG was also significant in an additive genetic model (effect = 18.792 mg/dL, *P* = 2.94*E* − 09). The TC genotype group showed higher serum TG levels (131.66 ± 86.23 mg/dL) than the TT genotype group (120.85 ± 69.71 mg/dL), and the CC genotype group showed the highest serum TG levels (166.8 ± 122.26 mg/dL). However, there were no significant differences in BMI, FBG, LDL-C, and total cholesterol in these genotype groups (Table 2).

Since it has been reported that gender is an important impact factor to the correlation of serum APOA5 with HDL-C and TG [26–28], we reanalyzed separately by gender. Interestingly, both in male and female subjects, the C allele had relations with higher levels of TG (in male effect = 30.208 mg/dL, *P* = 6.47*E* − 07; in female effect = 10.355 mg/dL, *P* = 0.002), but the minor allele had lowering effects on the levels of HDL-C only for males (effect = −2.124 mg/dL, *P* = 0.001; Table 3).

### 3.2. Association of *APOA5* −1131T>C Genotype with Clinical Parameters in SCGs

Since serum triglyceride (TG) concentrations reflect a complex interaction between multiple genetic and environmental factors, we hypothesized that the polymorphism of *APOA5* −1131T>C would affect serum TG levels differently as a risk factor among SCGs. To further define the association and relative risk of hypertriglyceridemia conferred by SCM that predispose to elevated plasma triglyceride TG concentrations, 1,619 individuals were classified into three constitutions groups and gender, as described in Section 2. The clinical characteristics of the three SCGs separated by gender were described in Table 4. There was no significant difference in the distribution of gender among the three SCGs by the Chi-square test (*P* = 0.132). We found statistically significant interconstitutional variations in BMI, FBG, HDL-C, LDL-C, total cholesterol, and TG (Table 4). Interestingly, the TE group displayed significantly elevated BMI, FBG, LDL-C, total cholesterol, and TG, and depressed HDL-C compared to the SY and SE groups.

The minor allele frequency of the *APOA5* −1131T>C was 0.291, 0.299, and 0.325 in SE, SY, and TE, respectively. There was no significant difference in the distribution of the *APOA5* −1131T>C genotype among the three SCGs by the Chi-square test (*P* = 0.133). The distribution of genotype was in the Hardy-Weinberg equilibrium although separated by SCGs. Interestingly, there was a significant association between the serum HDL-C levels and the *APOA5* −1131T>C genotype in SY and TE groups (Table 5). Subjects with the C allele in the SY and TE groups showed significant lower serum HDL-C levels than noncarriers of the C allele in an additive model (in SY, effect = −1.602 mg/dL, *P* = 0.03; in TE, effect = −1.659 mg/dL, *P* = 0.008). In addition to HDL-C, the impact of the *APOA5* −1131T>C genotype on the serum TG levels was also observed in the SY and TE groups, but not in the SE group (in SY, effect = 24.43 mg/dL, *P* = 3.36*E* − 06; in TE, effect = 17.155 mg/dL, *P* = 0.001). On the other hand, BMI, LDL-C, and total cholesterol were not significantly different according to genotypes among the SCGs.

## 4. Discussion

The polymorphism of *APOA5* −1131T>C has been suggested as a potential genetic factor involved in elevated serum triglyceride levels and a predictor of the risk and incidence of CVD [4, 5, 8]. In the present study, we investigated the roles of the *APOA5* −1131T>C polymorphism in serum lipid levels in the Korean population.

The C-allele minor frequency of our study population (0.3048) was consistent with the frequency previously reported for Korean subjects [29, 30]. Our study demonstrated a significant association between the *APOA5* −1131T>C polymorphism and serum TG and HDL-C levels (Table 2), reemphasizing that the *APOA5* −1131T>C polymorphism has a significant impact on both serum HDL-C and TG levels. Although there are still discrepant results regarding to the association of the *APOA5* −1131T>C with serum HDL-C levels [8, 10, 28, 31], interestingly, our study revealed that the HDL-C levels were significantly lowered through the per-allele carriages. The serum HDL-C levels of subjects with the CC genotype were 7.4% lower than those of subjects with the TT genotype.

Furthermore, it should be noted that we observed the impact of gender on genotype-phenotype response in serum HDL-C levels since the *APOA5* −1131T>C polymorphism was only significantly associated with serum HDL-C in male subjects, not in female subjects (Table 3). These results contradict the report that the HDL-C-lowering effect of the *APOA5* −1131T>C polymorphism is less in male than in female [31, 32], but is consistent with the results of a recent meta-analysis [13] in that the effect of variants in the *APOA5* gene on plasma HDL-C was more pronounced in males than in female subjects.

Several studies conducted with a large population have consistently shown that the *APOA5* −1131T>C polymorphism is strongly associated with quantitative differences in serum TG levels [8, 11, 15, 28–30]. Our results also indicated that the *APOA5* −1131T>C polymorphism is associated with elevated serum TG levels in the Korean populations investigated (Table 2), although the impact varied depending on gender (Table 3). The serum TG levels of TC and CC were 8.9% and 38% higher than noncarriers (TT) in total population, respectively. In males, the serum TG levels of TC and CC were 12.37% and 54.85% higher than noncarriers (TT), respectively, whereas, in females, the serum TG levels of TC and CC were 5.52% and 22.16% higher than noncarriers (TT), respectively, suggesting that the impact of the *APOA5* genotype on serum TG levels may be gender dependent.

One of the previously reported variants showing an association with TG [8], rs2266788 (1259T/C), was found to be tightly linked with rs180349 (A>T) SNP (*r* ^2^ = 0.85, *D*′ = 0.94) in HapMap Han Chinese and Japanese HapMap populations (HapMap-HCB and JPT) [33] via the Haploview program (version 4.2) whereas rs226679 is tightly linked with rs481843 SNP in Caucasian HapMap population (HapMap-CEU) [34]. The T allele of rs180349 was significantly associated with TC and TG in 979 Koreans by the affymetrix chip analysis (table not shown, effect = 5.39 mg/dL, *P* = 3.7*E* − 3 for TC; effect = 19.48 mg/dL, *P* = 6.12*E* − 6 for TG). This rs180349 SNP was also tightly linked with rs662799 (*APOA5* −1131T>C) based on HapMap HCB and JPT (*r* ^2^ = 0.69, *D*′ = 0.90). Therefore, the *APOA5* −1131T>C polymorphism may be in same haplotype block with 1259T/C polymorphism via rs180349 SNP, confirming the genetic effect of *APOA5* gene on TG in Koreans. There were no gender-specific associations with the SNP rs180349.

In our study, serum TG levels were higher in TE followed by SY and SE, but the HDL-C levels were reversed (Table 4), leading us to analyze the differences in allele frequency of the *APOA5* −1131T>C among the SCGs; but there were no statistically significant differences. However, multiple regression analysis showed that effect of *APOA5* −1131T>C polymorphism on serum HDL-C lowering and TG elevating were greater in the TE and SY groups than the SE group. It is interesting that these effects were significant only in the SY and TE groups not in the SE group.

These findings have a thread of connection with the theory of SCM that there are differences in the susceptibility to pathological conditions according to SCG [35]. Speculatively, it is suggested that the *APOA5 *−1131T>C polymorphism do not produce categorical dyslipidemia but functions as modifier for serum HDL-C and TG levels according to SCG. Moreover, these differential effects of *APOA5 *−1131T>C polymorphism on serum lipids levels are reminiscent of previous reports that the prevalence and relative risk of several chronic disease were found to differ across different SCGs and the SCG showed significant and independent association with diabetes mellitus [17, 35, 36]. The differences in the prevalence of decreasing serum HDL-C and elevating serum TG levels according to SCG together with *APOA5 *−1131T>C polymorphism suggest that SCG may act as a significant risk factor of hypo-HDL-C-emia and hypertriglyceridemia susceptibility. Moreover, the effect of rs180349 on TG levels were exclusively enriched in the group of SY type (table not shown, effect = 30.76 mg/dL, *P* = 2.23*E* − 5). Therefore, we believe that comprehensive knowledge of SCG and genotyping individuals for *APOA5* polymorphisms may be helpful in predicting disease susceptibility.

## 5. Conclusions

In conclusion, the association of *APOA5 *−1131T>C polymorphism with serum HDL-C and TG levels were clearly established in Korean subjects in the current study. In particular, the C allele of *APOA5 *−1131 was associated with lower serum HDL-C and higher TG in SY and TE but not in SE subjects. Therefore, we suggest that this genetic marker, together with SCG, may help in the identification of subjects who are highly susceptible to hypo-HDL-C-emia and hypertriglyceridemia, so that preventive care may be undertaken to reduce CVD.

## Figures and Tables

**Table 1:** Clinical and biochemical characteristics of the study subjects.

Variables	Mean ± SD			*P*-value
	Male (*n* = 595)	Female (*n* = 1024)	Total (*n* = 1619)	
Age (years)	47.38 ± 14.88	48.45 ± 14.95	48.06 ± 14.93	0.07
BMI (kg/m^2^)	24.02 ± 3.3	23.17 ± 3.38	23.49 ± 3.37	6.93*E*−06
FBG (mg/dL)	102.49 ± 30.87	96.45 ± 25.23	98.66 ± 27.57	6.63*E*−05
HDL-C (mg/dL)	41.82 ± 10.28	48.66 ± 11.98	46.15 ± 11.85	1.82*E*−31
LDL-C (mg/dL)	106.22 ± 29.03	106.85 ± 30.16	106.62 ± 29.74	0.51
Total cholesterol (mg/dL)	185.53 ± 33.79	189.65 ± 34.45	188.14 ± 34.26	0.01
TG (mg/dL)	149.97 ± 96.53	117.48 ± 72.61	129.38 ± 83.64	2.51*E*−14

Values are indicated as the mean ± standard deviation.

*P*-value: Student's *t*-test result.

Abbreviations: BMI, body mass index; FBG, fasting blood glucose; HDL-C, high-density lipoprotein cholesterol; LDL-C, low-density lipoprotein cholesterol; TG, triglyceride.

**Table 2:** Clinical and biochemical characteristics according to the *APOA5 *−1131T>C polymorphism and association of the *APOA5 *−1131T>C polymorphism with BMI, FBG, and serum lipid variables in the study population.

Variables	Mean ± SD			Multiple regression			
	TT (*n* = 765)	TC (*n* = 712)	CC (*n* = 142)	Slope	95% CI of slope		*P*-value
BMI (kg/m^2^)	23.6 ± 3.42	23.37 ± 3.31	23.93 ± 3.31	0.04	−0.211	0.291	0.755
FBG (mg/dL)	99.4 ± 29.95	98.02 ± 26.29	96.55 ± 18.37	−1.185	−3.245	0.875	0.259
HDL-C (mg/dL)	46.91 ± 11.68	45.76 ± 12.17	43.44 ± 10.33	−**1.602**	−2.481	−0.723	**3.61** **E**−**04**
LDL-C (mg/dL)	107.28 ± 28.47	105.49 ± 30.98	108.95 ± 28.76	−0.022	−2.247	2.203	0.984
Total cholesterol (mg/dL)	188.5 ± 32.68	186.87 ± 35.45	192.87 ± 35.39	0.845	−1.728	3.417	0.52
TG (mg/dL)	120.85 ± 69.71	131.66 ± 86.23	166.8 ± 122.26	**18.792**	12.616	24.968	**2.94** **E**−**09**

Values are indicated as the mean ± standard deviation.

*P*-value: multiple regression analysis result.

Bold indicates statistically significant (*P* < 0.05).

Abbreviations: CI, confidence interval; BMI, body mass index; FBG, fasting blood glucose HDL-C, high-density lipoprotein cholesterol; LDL-C, low-density lipoprotein cholesterol; TG, triglyceride.

**Table 3:** Association of the *APOA5 *−1131T>C polymorphism with BMI, FBG, and serum lipid variables according to gender in study population.

Variables	Gender	Mean ± SD			Multiple regression			
		TT	TC	CC	Slope	95% CI of slope		*P*-value
BMI (kg/m^2^)	Male	24.14 ± 3.41	23.84 ± 3.29	24.26 ± 2.98	−0.058	−0.469	0.353	0.781
	Female	23.3 ± 3.39	23.09 ± 3.3	23.72 ± 3.51	0.052	−0.26	0.363	0.744
FBG (mg/dL)	Male	103.81 ± 35.87	101.16 ± 25.92	98.65 ± 20.74	−2.149	−5.866	1.568	0.257
	Female	97.01 ± 25.91	96.12 ± 26.36	95.14 ± 16.58	−0.815	−3.231	1.601	0.508
HDL-C (mg/dL)	Male	42.82 ± 10.46	41.29 ± 10.31	38.32 ± 7.09	−**2.124**	−3.362	−0.887	**0.001**
	Female	49.13 ± 11.72	48.48 ± 12.42	46.88 ± 10.76	−0.987	−2.106	0.131	0.084
LDL-C (mg/dL)	Male	107.79 ± 28.19	104.48 ± 30.24	104.67 ± 27.97	−2.262	−5.882	1.358	0.22
	Female	107 ± 28.65	106.1 ± 31.44	111.82 ± 29.09	1.177	−1.615	3.969	0.408
Total cholesterol (mg/dL)	Male	186.3 ± 32.22	183.3 ± 35.19	188.67 ± 36.28	−0.481	−4.71	3.747	0.823
	Female	189.7 ± 32.89	189.04 ± 35.46	195.69 ± 34.71	1.62	−1.582	4.822	0.321
TG (mg/dL)	Male	135.58 ± 71.94	152.35 ± 95.84	209.95 ± 163.59	**30.208**	18.416	42	**6.47** **E**−**07**
	Female	112.86 ± 67.2	119.1 ± 77.28	137.87 ± 71.68	**10.355**	3.752	16.958	**0.002**

Values are indicated as the mean ± standard deviation.

*P*-value: multiple regression analysis result.

Bold indicates statistically significant (*P* < 0.05).

Abbreviations: CI, confidence interval; BMI, body mass index; FBG, fasting blood glucose HDL-C, high-density lipoprotein cholesterol; LDL-C, low-density lipoprotein cholesterol; TG, triglyceride.

**Table 4:** Clinical and biochemical characteristics of the study subjects according to SCG.

Variables	Mean ± SD												*P*-value^a^	*P*-value^b^
	SY				SE				TE					
	Male (*n* = 186)	Female (*n* = 360)	Total (*n* = 546)	*P*-value^c^	Male (*n* = 141)	Female (*n* = 264)	Total (*n* = 405)	*P*-value^c^	Male (*n* = 268)	Female (*n* = 400)	Total (*n* = 668)	*P*-value^c^		
Age (years)	49.39 ± 14.07	46.91 ± 14.65	47.76 ± 14.49	0.058	44.14 ± 15.89	46.65 ± 14.63	45.77 ± 15.11	0.111	47.32 ± 14.77	51.44 ± 14.86	49.78 ± 14.95	4.62*E*−04	8.55*E*−05	—
BMI (kg/m^2^)	23.68 ± 3.03	22.4 ± 2.7	22.84 ± 2.88	7.07*E*−07	21.52 ± 2.78	21.33 ± 2.56	21.4 ± 2.63	0.48	25.57 ± 2.86	25.26 ± 3.31	25.39 ± 3.14	0.208	4.09*E*−97	7.63*E*−92
FBG (mg/dL)	104.25 ± 31.39	96.46 ± 28.72	99.11 ± 29.86	0.004	97.09 ± 25.15	91.4 ± 13.4	93.38 ± 18.53	0.003	103.28 ± 32.09	99.82 ± 27.79	101.21 ± 29.62	0.139	2.92*E*−05	7.58*E*−04
HDL-C (mg/dL)	41.81 ± 10.81	49.21 ± 11.78	46.67 ± 11.98	2.92*E*−12	45.05 ± 10.09	51.34 ± 11.99	49.15 ± 11.74	1.84*E*−07	39.85 ± 9.34	46.41 ± 11.69	43.78 ± 11.27	5.49*E*−14	1.19*E*−12	1.57*E*−09
LDL-C (mg/dL)	103.41 ± 29.5	105.76 ± 29.37	104.96 ± 29.41	0.378	101.81 ± 26.97	101.5 ± 28.06	101.61 ± 27.65	0.916	109.99 ± 29.53	111.77 ± 30.95	111.05 ± 30.38	0.457	6.58*E*−07	1.08*E*−05
Total cholesterol (mg/dL)	183.85 ± 33.74	188.81 ± 33.65	187.12 ± 33.73	0.104	179.27 ± 32.17	183.86 ± 31.48	182.26 ± 31.76	0.166	189.19 ± 34.67	194.9 ± 35.69	192.61 ± 35.37	0.041	5.97*E*−06	3.77*E*−05
TG (mg/dL)	158.58 ± 104.8	114.1 ± 67.92	129.25 ± 84.93	4.01*E*−09	111.72 ± 58.15	96.5 ± 49.36	101.8 ± 53.02	0.006	164.82 ± 102.87	134.77 ± 84.02	146.83 ± 93.14	3.98*E*−05	7.00*E*−17	1.11*E*−13

Values are indicated as the mean ± standard deviation.

*P*-value^a^: analysis of variance (ANOVA) result.

*P*-value^b^: analysis of covariance (ANCOVA) result.

*P*-value^c^: Student's *t*-test result.

Abbreviations: SY, So-Yang; SE, So-Eum; TE, Tae-Eum; BMI, body mass index; FBG, fasting blood glucose; HDL-C, high-density lipoprotein cholesterol; LDL-C, low-density lipoprotein cholesterol; TG, triglyceride.

**Table 5:** Association of the *APOA5 *−1131T>C polymorphism with BMI, FBG, and serum lipid variables according to SCG in study population.

Variables	Constitution	Mean ± SD			Multiple regression			
		TT	TC	CC	Slope	95% CI of slope		*P*-value
BMI (kg/m^2^)	SY	22.81 ± 3.12	22.77 ± 2.65	23.25 ± 2.66	0.132	−0.224	0.489	0.465
	SE	21.56 ± 2.7	21.21 ± 2.5	21.63 ± 3.13	−0.098	−0.518	0.322	0.647
	TE	25.42 ± 3.12	25.35 ± 3.17	25.38 ± 3.22	−0.049	−0.412	0.314	0.79

FBG (mg/dL)	SY	100.23 ± 32.1	98.06 ± 29.59	98.31 ± 17.52	−1.256	−5.022	2.51	0.513
	SE	93.4 ± 20.04	93.34 ± 17.35	93.54 ± 16.09	0.112	−2.862	3.086	0.941
	TE	102.25 ± 32.46	101.17 ± 28.02	96.19 ± 19.92	−2.45	−5.826	0.926	0.155

HDL-C (mg/dL)	SY	47.62 ± 11.43	46.21 ± 12.93	44.26 ± 9.77	−**1.602**	−3.043	−0.16	**0.03**
	SE	49.24 ± 12.07	49.42 ± 11.55	46.29 ± 10.65	−0.537	−2.368	1.294	0.564
	TE	44.97 ± 11.35	42.91 ± 11.24	41.69 ± 10.5	−**1.659**	−2.883	−0.436	**0.008**

LDL-C (mg/dL)	SY	106.75 ± 29.92	103.34 ± 28.71	103.39 ± 29.99	−1.873	−5.562	1.816	0.319
	SE	100.35 ± 26.6	101.7 ± 28.43	110.71 ± 28.78	3.957	−0.482	8.396	0.081
	TE	111.76 ± 27.56	109.82 ± 33.88	112.98 ± 27.36	−0.37	−3.9	3.159	0.837

Total cholesterol (mg/dL)	SY	188.63 ± 33.64	184.87 ± 33.12	189.57 ± 36.75	−0.591	−4.836	3.654	0.785
	SE	180.76 ± 31.32	182.46 ± 32.27	192.25 ± 30.32	4.717	−0.395	9.829	0.07
	TE	192.94 ± 31.91	191.51 ± 38.77	195.89 ± 36.24	0.419	−3.683	4.52	0.841

TG (mg/dL)	SY	117.68 ± 69.29	130.47 ± 83.47	179.48 ± 130.64	**24.43**	14.212	34.648	**3.36** **E**−**06**
	SE	98.86 ± 51.96	102.62 ± 50.78	118.08 ± 74.47	7.99	−0.183	16.163	0.055
	TE	136.31 ± 75.28	152.41 ± 100.8	174.38 ± 126.37	**17.155**	6.696	27.614	**0.001**

Values are indicated as the mean ± standard deviation.

*P*-value: multiple regression analysis result.

Bold indicates statistically significant (*P* < 0.05).

Abbreviations: CI, confidence interval; SY, So-Yang; SE, So-Eum; TE, Tae-Eum; BMI, body mass index; FBG, fasting blood glucose; HDL-C, high-density lipoprotein cholesterol; LDL-C, low-density lipoprotein cholesterol; TG, triglyceride.

## Abbreviations

SCM: Sasang constitutional medicine
SCG: Sasang constitution group
SY: So-Yang
SE: So-Eum
TE: Tae-Eum
BMI: Body mass index
TG: Triglyceride
FBG: Fasting blood glucose
HDL-C: High-density lipoprotein cholesterol
LDL-C: Low-density lipoprotein cholesterol.
